# Complete Molar Pregnancy With Hyperthyroidism, High-Output Heart Failure and Peptoniphilus Species Bacteremia: A Case Report

**DOI:** 10.7759/cureus.111523

**Published:** 2026-06-25

**Authors:** Louise Mees, Jolan Kint, Pascale Abrams

**Affiliations:** 1 Internal Medicine, Antwerp University Hospital, Antwerp, BEL; 2 Endocrinology and Metabolism, Ziekenhuis aan de Stroom (ZAS) Vincentius, Antwerp, BEL

**Keywords:** high-output heart failure, hyperthyroidism, invasive hydatidiform mole, molar pregnancy, peptoniphilus species

## Abstract

A complete molar pregnancy is a rare but dangerous complication of pregnancy. Early diagnosis is crucial due to the potential harmful consequences, especially if recognized late. Complications include the malignant potential, hyperthyroidism, possible preeclampsia, and ovarian cysts. Here, we report a case study in which a 47-year-old woman with no medical history presented with heart failure and a systemic infection. Upon further investigation, she was found to have significant hyperthyroidism due to thyroid stimulation from a very high human chorionic gonadotropin (hCG) level, in the presence of a molar pregnancy. Furthermore, she was found to have bacteremia with Peptoniphilus species, typically present in the vaginal flora. This germ has not been previously demonstrated as a pathogen in the context of a molar pregnancy.

## Introduction

A molar pregnancy is a trophoblastic disease originating in the placenta, resulting from hyperplasia due to overexpression of paternal genes. A molar pregnancy can be complete (46XX, 46XY) or partial (69XXX, 69XXY, 69XYY). The incidence of a molar pregnancy in North America and Europe is one to one point five per 1000. It is not primarily a malignancy, but a premalignant condition. The risk of malignant development is 15 to 20 percent for a complete molar pregnancy and one to five percent for a partial molar pregnancy [1].

A molar pregnancy can induce secondary hyperthyroidism due to excess human chorionic gonadotropin (hCG) (a hormone with thyroid-stimulating properties) production [2-5]. In rare cases, this hyperthyroidism can lead to high-output heart failure due to functional changes in both the peripheral circulation and the myocardium [6,7].

Peptoniphilus species, belonging to the group of Gram-positive anaerobic cocci, is known as a commensal in the gastrointestinal and vaginal flora, but in some cases also appears to be relevant as a pathogen [8,9].

## Case presentation

A 47-year-old woman without any noticeable medical history presented at the emergency department with palpitations for one week and chills for two days. Further history taking revealed dysuria and anorexia, along with an eight kg weight loss in one month. Clinical examination revealed a febrile woman (40.1 °C) with severe hypertension (180/75 mmHg) and a rapid, regular pulse of 140 bpm. She also had signs of acute heart failure, with markedly congested jugular veins and peripheral pitting edema. The ECG revealed sinus tachycardia (Figure 1). A chest X-ray revealed signs of hilar congestion and blunted costodiaphragmatic recesses (Figure 2). A blood count revealed an inflammatory blood count with leukocytosis and an elevated CRP of 65 mg/dL. N-terminal pro B-type natriuretic peptide (NT-proBNP) is found to be nearly 10,000 ng/L with a non-evolving troponinemia of 600 ng/L (Table 1). Urine culture shows significant pyuria with 5149 white blood cells per field, with a normal range below 25 white blood cells per field.

**Figure 1:**
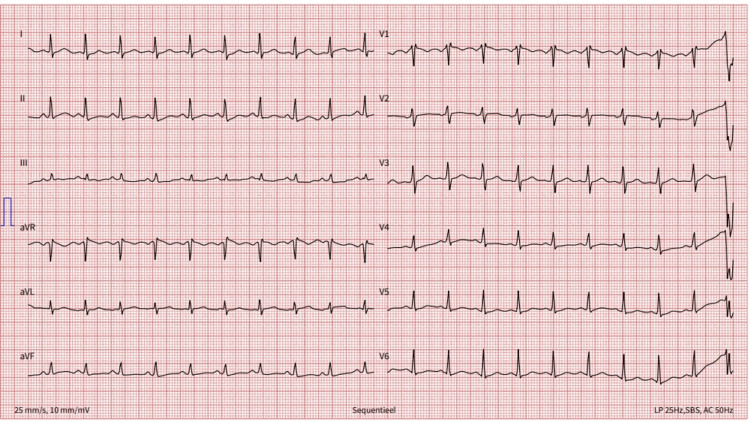
ECG ECG showing sinus tachycardia at 118 beats per minute.

**Figure 2:**
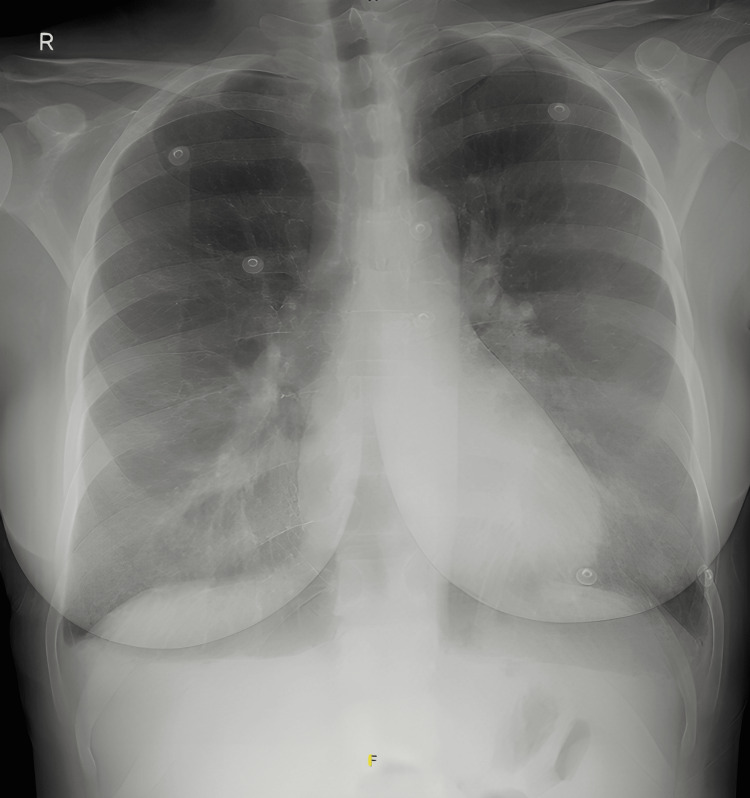
Chest X-ray Chest X-ray shows hilar congestion and blunted costodiaphragmatic recesses bilaterally.

After hemocultures were taken, antibiotics were started in the form of a single dose of amikacin, followed by amoxicillin-clavulanic acid intravenously. 

Further workup during hospitalization revealed clinical hyperthyroidism with a TSH lower than 0.01mE/L, free T4 of 44 pmol/L, free T3 of 20pmol/L, and negative TSH receptor antibodies. The hCG level was more than 1.8 million U/L (Table 1). The last menstruation was eight weeks ago, but they are known to be irregular for the patient. Transthoracic ultrasound revealed HFpEF (heart failure with preserved ejection fraction) without any other structural heart disease.

**Table 1:** Laboratory findings at diagnosis NT-proBNP: N-terminal pro B-type natriuretic peptide; hCG: human chorionic gonadotropin

Laboratory findings	Observed values	Reference ranges
Leucocytes	14.43 x 10^9/L	3.80-9.39 x 10^9/L
Neutrophils	13.20 x10^9/L	1.66-5.92 x 10^9/L
CRP (C-reactive protein)	65 mg/dL	< 4 mg/dL
Troponins	623.4 ng/L	< 14 ng/L
NT-proBNP	9686.9 pg/mL	<300 pg/mL
TSH	<0.01 mU/L	0.35-4.94 mU/L
Free T4	43.74 pmol/L	9.01-19.05 pmol/L
Free T3	19.98 pmol/L	3.10-6.80 pmol/L
hCG	1847182 U/L	< 6 U/L

After a few days, the urine culture revealed amoxicillin-clavulanic acid-sensitive E. coli. Two sets of hemocultures showed growth of omnisensitive Peptoniphilus species. Vaginal ultrasound confirmed the presence of a mole (Figure 3). 

**Figure 3:**
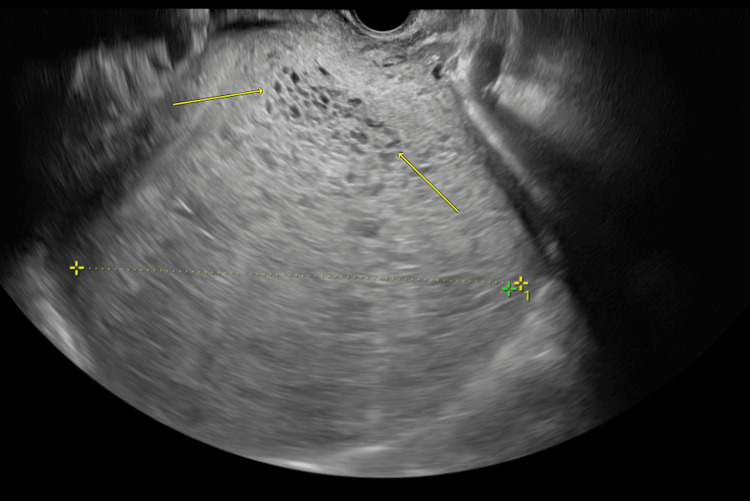
Transvaginal ultrasound Transvaginal ultrasound shows a vesicular mass in the cavum uteri, suggestive of a molar pregnancy, as shown between the arrows.

The patient underwent aspiration curettage for this purpose. Anatomical pathological examination revealed a complete hydatidiform mole without signs of choriocarcinoma. PTU (propylthiouracil) was initiated at a dose of 50mg twice daily, along with a sartan and an SGLT2 inhibitor. A follow-up cardiac ultrasound after a few weeks showed recovery of the HFpEF pattern.

Weekly follow-up of the hCG level was also provided. However, because a plateau was reached after several weeks (Figure 4 and Table 2), a new vaginal ultrasound was performed, which revealed uterine residual tissue with increased flow, suggestive of an invasive mole (Figure 5). A subsequent chest and abdomen CT confirmed the suspicion of a tumorous process in the uterus with metastases in both lungs (Figure 6).

**Figure 4:**
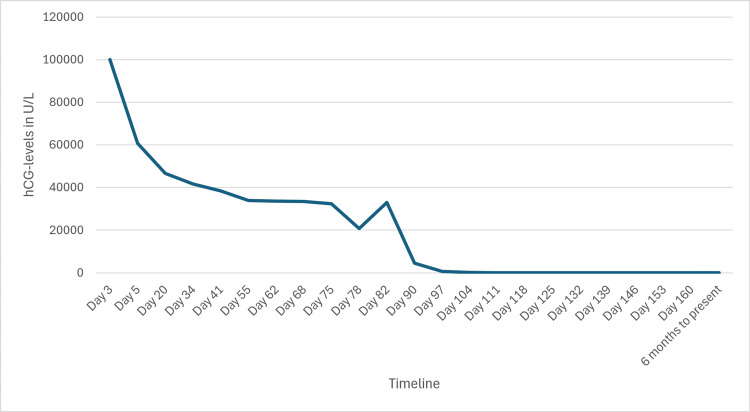
Graphic representation of the hCG levels starting after curettage (day 0) Graphic representation showing the evolution of the hCG levels chronologically. On day 0, the curettage was performed. Chemotherapy was initiated on day 76. hCG: human chorionic gonadotropin

**Table 2:** hCG levels in chronological order Day 0 is the day of diagnosis and curettage. Reference range of hCG: <6 U/L hCG: human chorionic gonadotropin

Timeline	hCG level in U/L
Day 0	1847182
Day 3	100081
Day 5	60755
Day 20	46596
Day 34	41691
Day 41	38479
Day 55	33943
Day 62	33562
Day 68	33453
Day 75	32411
Day 78	20771
Day 82	32949
Day 90	4497
Day 97	608
Day 104	155
Day 111	74
Day 118	37
Day 125	26
Day 132	17
Day 139	12
Day 146	10
Day 153	8
Day 160	6
6 months to present	< 6

**Figure 5:**
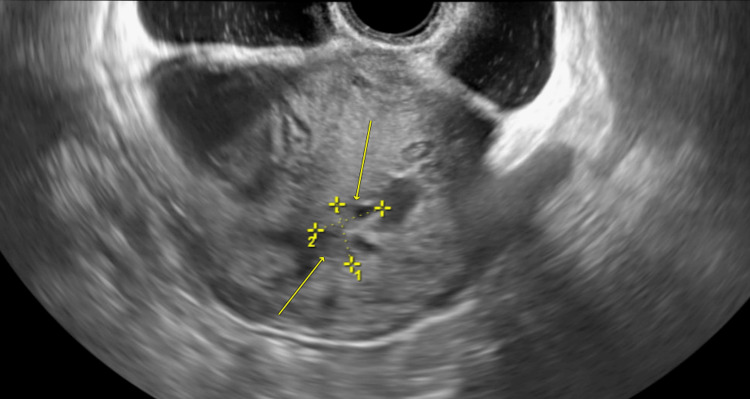
Follow-up transvaginal ultrasound 10 weeks after initial diagnosis Transvaginal ultrasound showing anechogenic zones (between the arrows) with increased vascularity in the fundus of the uterus, suggestive of an invasive mole.

**Figure 6:**
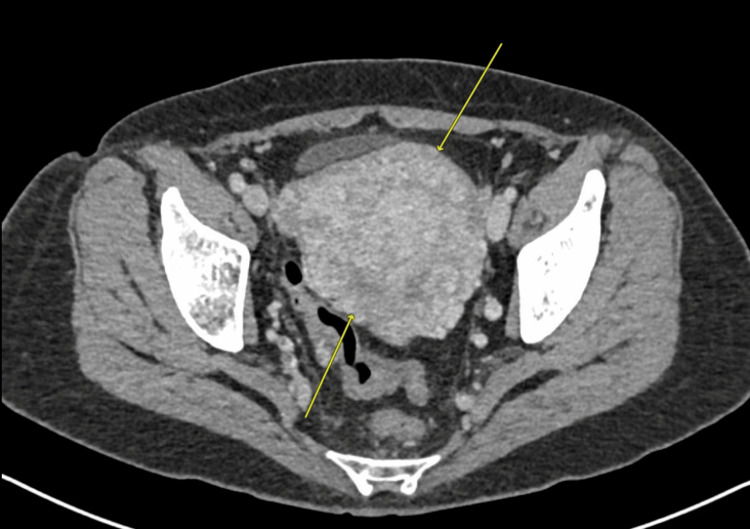
Abdominal CT Abdominal CT showing a large mass in the cavum uteri (between the arrows, measuring about 91mm x 83mm), suggestive of a hydatidiform mole.

Methotrexate was initiated, followed by a few weeks of escalation to an EMA-CO chemotherapy regimen (consisting of etoposide, methotrexate, actinomycin, cyclophosphamide, and vincristine), during which the hCG level normalized. The patient ultimately underwent a total hysterectomy with bilateral salpingo-oophorectomy nine months after the initial diagnosis.

## Discussion

A molar pregnancy is a trophoblastic disease originating in the placenta, resulting from hyperplasia due to overexpression of paternal genes. A molar pregnancy can be complete (46XX, 46XY) or partial (69XXX, 69XXY, 69XYY). Only the partial form is compatible with life. It is not a malignancy, but a premalignant condition. When it evolves into a malignancy, it is called gestational trophoblastic neoplasia, a group consisting of an invasive mole, choriocarcinoma, a placental site trophoblastic tumor, and an epithelioid trophoblastic tumor. The risk of malignancy is up to five percent for a partial molar pregnancy and up to 20 percent for a complete molar pregnancy. The incidence of molar pregnancies in North America and Europe is between one and one point five per 1000. Risk factors for molar pregnancies include extreme maternal age (>35 years or <20 years), multiparity, use of oral contraceptives, and previous molar pregnancies. The hCG level often rises above 100,000 U/L. Other complications include theca lutein cysts due to ovarian hyperstimulation, early-onset preeclampsia (<20 weeks), and hyperthyroidism [1].

Hyperthyroidism in molar pregnancies occurs due to the characteristic excess of hCG production. This hCG has intrinsic thyroid-stimulating activity due to the similarity between TSH and hCG. This molecular mimicry arises because both glycoprotein hormones share a similar alpha-subunit [3]. hCG's action on the TSH receptor stimulates the thyroid gland to produce T3 and T4, resulting in TSH suppression. The degree of thyroid stimulation and clinical signs of hyperthyroidism are directly correlated with hCG levels. hCG acts approximately 4000 times less potently on the TSH receptor, implying that massive hCG levels are required for a spillover effect. However, the beta-hCG produced by hydatidiform moles has a stronger thyrotropic activity than classic beta-hCG due to its reduced sialylation. Values ​​above 200,000 mIU/L suppress TSH in 67% of cases; at hCG levels above 400,000 mIU/L, TSH suppression occurs in 100% of cases.

Hyperthyroidism in molar pregnancies is reported to occur in 25-64% of cases. Up to five percent of molar pregnancies present with clinical signs of hyperthyroidism [2-4,10,11]. The classic symptoms of hyperthyroidism are shown in Table 3.

**Table 3:** Classical symptoms of hyperthyroidism Adapted from the text in [4]

Organ system	Classical symptoms
Skin	Heat intolerance, sweating, onycholysis, hyperpigmentation, pruritus, urticaria, vitiligo, alopecia areata, thinning hair
Eyes	Exophthalmia
Cardiovascular	Increased pulse, atrial fibrillation, systolic hypertension
Bone	Osteoporosis
Respiratory	Dyspnea
Gastro-intestinal	Weight loss, anorexia, hyperdefecation due to increased intestinal motility, diarrhea, steatorrhea
Urinary	Nocturia, enuresis, increased urinary frequency
Neuropsychiatric	Tremor, agitation, depression, psychosis, anxiety, erratic, irritable, insomnia

In some cases, hyperthyroidism can lead to high-output heart failure. This is reported to affect six percent of patients with hyperthyroidism. Hemodynamically, this can be explained by functional changes in both the peripheral circulation and the myocardium. These include an increase in circulating blood volume and a decrease in systemic vascular resistance. This results in increased preload and decreased afterload, which in turn causes an increase in heart rate and inotropy (in the form of increased stroke volume). The result is a hyperdynamic circulation that can lead to clinical heart failure [6,7,12-16].

The most extreme manifestation of hyperthyroidism is thyroid storm. The clinical presentation of this condition can be classified according to Burch and Wartofsky, as shown in Table 4. A fulminant thyroid storm has a mortality rate of up to 15% [1,10,17,18].

**Table 4:** Classification of thyroid storm according to Burch and Wartofsky Adapted from the text in [18]

Classification of thyroid storm according to Burch and Wartofsky		
Temperature	37.2 - 37.7°C	5
	37.8 - 38.2°C	10
	38.3 - 38.8°C	15
	38.9 - 39.4°C	20
	39.4 - 39.9°C	25
	>40.0°C	30
Neurological symptoms	Mild (agitation)	10
	Moderate (delirium, psychosis, lethargy)	20
	Severe (insult, coma)	30
Tachycardia	99 - 109	5
	110 - 119	10
	120 - 129	15
	130 - 139	20
	>140	25
	Atrial fibrillation	10
Heart failure	Mild (edema)	5
	Moderate (crepitations on clinical exam)	10
	Severe (lung edema)	15
Gastro-intestinal	Moderate (diarrhea, nausea/vomiting, abdominal pain)	10
	Severe (icterus)	20
Relevant medical history	Negative	0
	Positive	10
Score	Thyroid storm unlikely	<25
	Possible thyroid storm	25-45
	Thyroid storm	>45

In retrospect, our patient met the criteria for a thyroid storm according to Burch and Wartofsky; however, given the presence of bacteremia, there is a secondary explanation for the high score. Another argument against a thyroid storm is that the patient's hemodynamics partially improved after starting antibiotics.

The possibility of an impending thyroid storm and possible accompanying high-output heart failure should be considered in the perioperative period. Premedication with PTU, beta-blockade, iodine, steroids, and monitoring (peri- and postoperatively) play an important role in this [19,20].

Peptoniphilus species, belonging to the Gram-positive anaerobic cocci group, are known to be commensal in the gastrointestinal and vaginal flora. However, in some cases, Peptoniphilus also appears to be relevant as a pathogen in certain infections. This is usually the case as part of a polymicrobial infection, and only very rarely as a monopathogen. This primarily concerns skin and soft tissue infections such as intra-abdominal infections (chorioamnionitis, ovarian or retroperitoneal abscesses, etc.) and, in rare cases, bacteremia. To date, it is not fully understood how this commensal enters the bloodstream. Articles suggest invasive procedures (e.g., IUD, colposcopy, etc.), the presence of erosive tumors, or immunosuppressive status as contributing factors. Wang et al. describe a case of a young woman who died from Peptoniphilus sepsis. The presence and clinical relevance of Peptoniphilus species are clearly underestimated to date [8,9]. In our patient, no other etiology was found for the bacteremia with Peptoniphilus species, other than the presence of a molar pregnancy.

## Conclusions

A molar pregnancy is a serious but rare condition that can impact various organ systems. Our case involves a woman who developed hyperthyroidism with concomitant heart failure because of a complete molar pregnancy. She also had Peptoniphilus bacteremia, a rare finding. A new diagnosis of heart failure at a young age should include sufficiently comprehensive screening and diagnostics for the underlying etiology. With early diagnosis and adequate treatment, this condition is completely curable.
